# Profiling bile acids in the stools of humans and animal models of cystic fibrosis

**DOI:** 10.1128/spectrum.01451-25

**Published:** 2025-09-16

**Authors:** Melissa M. Carmichael, Rebecca A. Valls, Shannon Soucy, Julie Sanville, Juliette Madan, Sarvesh V. Surve, Mark S. Sundrud, George A. O’Toole

**Affiliations:** 1 Department of Microbiology and Immunology, Geisel School of Medicine at Dartmouth 12285, Hanover, New Hampshire, USA; 2 Walter and Carole Young Center for Digestive Health, Dartmouth Health 4456, Lebanon, New Hampshire, USA; 3 Department of Medicine, Dartmouth Health 4456, Lebanon, New Hampshire, USA; 4 Department of Biomedical Data Science, Geisel School of Medicine, Hanover, New Hampshire, USA; 5 Department of Pediatrics, Dartmouth Health 4456, Lebanon, New Hampshire, USA; 6 Department of Psychiatry, Dartmouth Health 4456, Lebanon, New Hampshire, USA; 7 Department of Epidemiology, Geisel School of Medicine at Dartmouth 12285, Hanover, New Hampshire, USA; 8 Department of Quantitative Biomedical Data Science, Geisel School of Medicine at Dartmouth 12285, Hanover, New Hampshire, USA; 9 Dartmouth Cancer Center, Dartmouth Health 4456, Lebanon, New Hampshire, USA; University of Nebraska-Lincoln, Lincoln, Nebraska, USA

**Keywords:** bile acids, cystic fibrosis, human, mouse, ferret

## Abstract

Cystic fibrosis (CF) is associated with aberrant bile acid (BA) metabolism. As little is known about BA in children with CF (cwCF), we performed both comprehensive (*n* = 89) and focused (*n* = 21) BA profiling in stool of children with or without CF. Our results reveal select BA species and metabolites are significantly different between cwCF and nonCF controls. Focused BA profiling revealed a significant increase in total BA levels and selected changes in a subset of BA classes for cwCF. Matched bacterial metagenomic analyses showed no change in alpha-diversity between groups in this small cohort, at odds with previous studies, whereas changes in relative abundance of *Bacteroidetes* (lower in cwCF) phylum are consistent with prior reports. A trend was noted toward reduced abundance of *bsh* gene families, a key rate-limiting enzyme required for bacterial synthesis of secondary BAs, in cwCF. Observed modest changes in both BAs and microbial BA metabolism-related gene abundances may suggest a possible combination of defects in host and microbial BA metabolic pathways in cwCF. Fecal BA profiles from both ferret and mouse CF models showed significant differences from human BA profiles, and while the ferret model reproduced significant differences between CF and nonCF animals, the nonCF animals showed higher levels of BA (opposite of what is observed in humans), indicating that neither model recapitulated BA in stool in the context of CF. Together, these results provide new insights into CF-related BA dysmetabolism in cwCF and highlight limitations of CF animal models for BA functional studies.

**IMPORTANCE:**

Changes in the abundance and/or composition of intestinal BAs may contribute to dysbiosis and altered gastrointestinal physiology in CF. Here, we report shifts in select fecal BA classes and species for cwCF. Matched metagenomic analysis suggests possible defects in both host intestinal BA absorption and gut microbial BA metabolism. Additional analyses of mouse and ferret CF stool for BA composition suggest great care must be taken when interpreting BA functional studies using these animal models. Together, this work lays technical and conceptual foundations for interrogating BA-microbe interactions in cwCF.

## INTRODUCTION

Cystic fibrosis (CF) is a hereditary disease caused by mutations in the gene encoding the cystic fibrosis transmembrane conductance regulator (CFTR) protein. Disruption of CFTR function results in alterations in the secretion of chloride and bicarbonate causing an imbalance in the hydration of mucosal surfaces, and accumulation of abnormally thick, viscous mucus in the lungs, as well as biliary and intestinal tracts (1–3). Whereas pulmonary disease dominates the adult population of persons with CF (pwCF), gastrointestinal (GI) complications are an important cause of morbidity for pwCF early in life. Infants and children with CF (cwCF) can experience an array of GI symptoms including meconium ileus, small intestinal bacterial overgrowth, and nutrient malabsorption (1, 3–6).

From an early age, cwCF exhibit a decrease in α-diversity, as evidenced by fecal 16S rRNA amplicon library and metagenomic sequencing (7–11). Literature indicates the persistence of this dysbiosis into adulthood among pwCF (3, 5, 12, 13). Notable taxonomic differences observed in cwCF include a reduction in specific immune-modulating bacterial genera in the phyla Bacteroidetes (i.e., *Bacteroides* spp.), which is positively associated with gut health, and an increase in pathogenic Proteobacteria (i.e., *Escherichia coli*) (7–11). These shifts in colonic microbial communities are likely driven by changes in the physiological features of the intestinal environment (2, 14). The physiological features of the CF colon can be characterized by excess mucus and fat content, acidic pH, inflammation, antibiotic perturbations, as well as altered bile acids (BA) (15–18).

BAs play key roles in shaping gut microbial colonization and function, acting as detergents and supporting the absorption of fats by intestinal enterocytes (19–21). While the liver is responsible for the production of primary BAs, those that escape intestinal absorption in the distal small intestine (i.e., ileum) become increasingly subject to modification by microbiota in the large intestine. The products of bacterial BA metabolism (i.e., unconjugated BA and secondary BA species and metabolites) dramatically increase the structural and functional diversity of BAs in enterohepatic circulation (19, 21). BAs have direct anti-microbial functions, impacting susceptible bacteria in both a bacteriostatic and bactericidal fashion, via disruption of bacterial membranes (20, 21). Consequently, the modification of BAs is an essential microbial defense mechanism (20, 21). For example, when tested against *Lactobacillus* and *Bifidobacterium*, primary conjugated BAs were shown to disrupt membranes in a dose-dependent manner, while unconjugated BAs exert a greater reduction in viability than their conjugated counterparts (19, 22).

Among the hallmarks of GI complications in pwCF, as well as in murine models, is a reported threefold increase in fecal BA excretion (20, 21, 23–25). Enterohepatic circulation of BAs is a tightly regulated system in which ~95% of total BAs are reabsorbed in the ileum and the remaining ~5% pass through the colon and are excreted via the feces (21). In a healthy individual, the secondary BAs lithocholic acid (LCA) and deoxycholic acid (DCA) dominate fecal profiles (26). Previous studies suggest that impaired BA homeostasis manifests as BA malabsorption and an increased cholic acid (CA) to chenodeoxycholic acid (CDCA) ratio among duodenal BAs in adult pwCF (20, 23, 24). Two studies have been performed using samples from children ages ranging from 2 years to 20 years (25, 27). However, no studies have focused on BA homeostasis in cwCF and on the relationship between BAs and gut microbial communities in the CF gut. Previous studies also profiled only a fraction—typically a small handful—of the estimated hundreds of BA species now recognized in the human intestinal tract. We address these knowledge gaps here.

Improved animal models have emerged as key tools for deciphering mechanistic links between gut microbiota and disease in CF (2, 28). Thus, we also sought to examine the relevance of BA profiles within two common and notable CF animal models—ferret and mouse. We show that stool BA profiles in ferrets and mice with CF do not parallel those of cwCF although there is a difference in BA profiles in ferrets with and without CF (although this difference is the opposite of what is noted in humans). Hence, caution must be taken when selecting animal models for the study of BA functions in the context of CF.

Altogether, these data provide key new insights for understanding relationships between BAs and microbial communities in the gut of cwCF, as well as for developing relevant *in vitro* and *in vivo* model systems to disentangle complex relationships between intestinal BAs and microbiota. These investigations also lay the groundwork for clarifying interventions leveraging the complex micro- and macro-environment of the intestine in cwCF with the goal of optimizing systemic health.

## RESULTS

### CF-related alterations in the fecal bile acid metabolome among children

We first surveyed a comprehensive panel of 89 BA species and related metabolites in feces (stool) for 15 individual children with cystic fibrosis (cwCF) and 15 healthy controls (Fig. S1A) using ultra performance liquid chromatography mass spectrometry (UPLC-MS/MS). Fecal samples capture the fraction of BAs that escape ileal absorption and are metabolized in the large intestine. Eighty-four of 89 species examined displayed values > 0 in at least one of the samples (CF or nonCF) and were, thus, considered detectable. These BA species and metabolites clustered into seven general classes based on biosynthetic origins (Fig. S1B and C): (i) primary conjugated BAs (primary cBA), which are BAs synthesized and secreted by the liver at homeostasis; (ii) primary unconjugated BA (primary uBA), which are products of bacterial primary cBA deconjugation; (iii) secondary conjugated BAs (secondary cBA), which are secondary uBAs re-conjugated in hepatocytes; (iv) secondary unconjugated BA (secondary uBA), which are products generated from primary uBAs via bacterial metabolism; (v) secondary metabolites, which are more extensively modified secondary uBAs produced by bacterial metabolism; (vi) hepatic detoxification products, including sulfate and glucuronide conjugates produced to limit BA toxicity and promote clearance; and (vii) synthetic, intermediates, and atypical BAs, which are normally secreted in trace amounts by healthy livers, but can increase during active liver disease (29).

**Fig 1:**
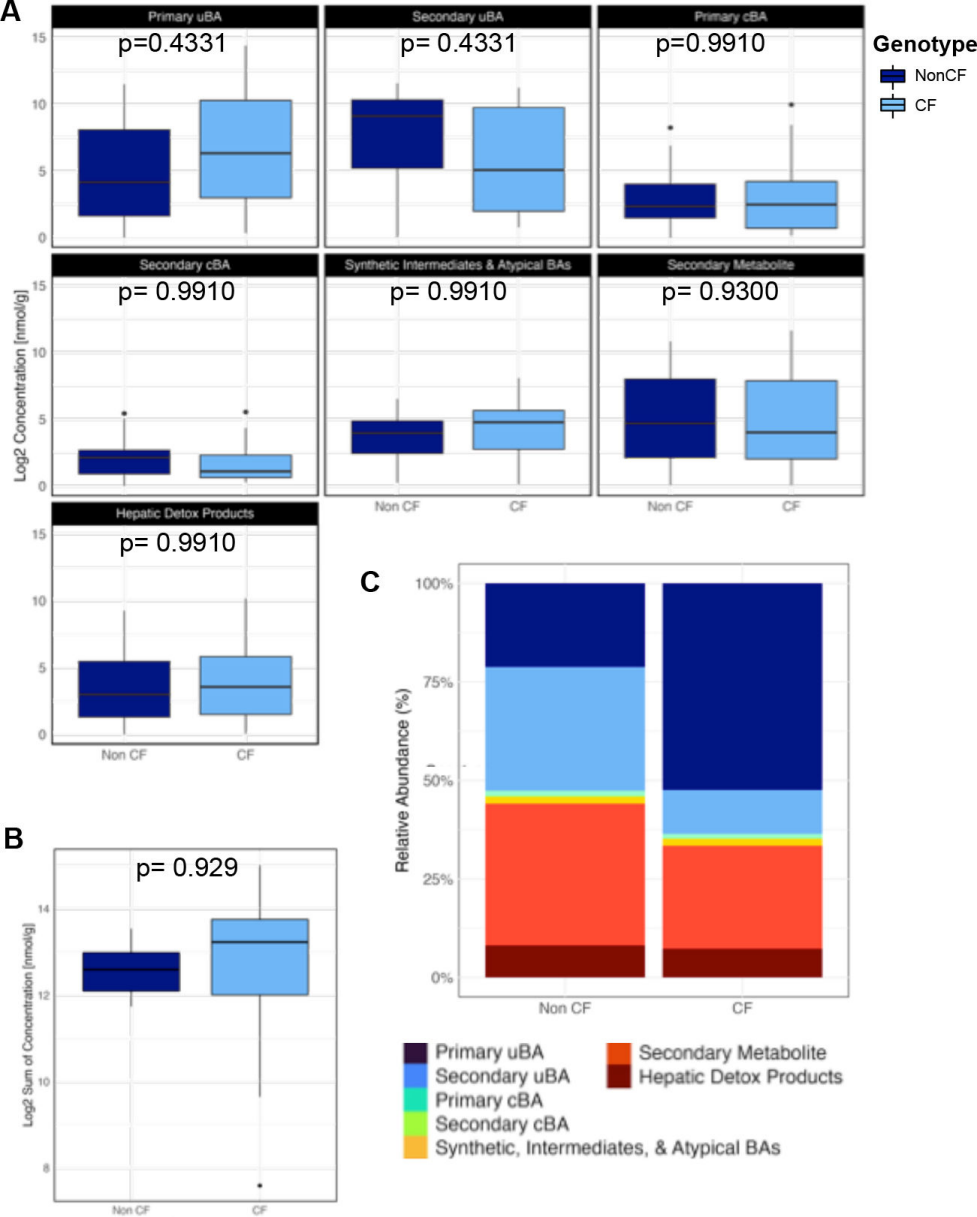
Summary of comprehensive BA profiles from human stool samples. (**A**) Box plots comparing log2 transformed concentration for each of the seven classes from human fecal samples (*n* = 15) of each genotype (CF and nonCF). Statistical significance was determined by linear model, which is summarized in Tables S1 and S2. *P* values are shown. (**B**) Total bile acid concentration (log2 transformed) for each sample from the same human fecal samples in Panel A (*n* = 15) of each genotype (CF and nonCF). Linear model does not show significance (*P*-value = 0.929). *P* value is shown. (**C**) Relative abundance of functional groups between genotypes (CF and nonCF) in human samples.

Comparison of log-transformed total concentration for each of the seven BA classes between CF and nonCF showed BAs derived from hepatocytes—primary uBAs, synthetic intermediates and atypical BAs primary cBAs, and hepatic detoxification products—showed a non-significant elevation in stool of cwCF vs controls, while those produced directly via microbial metabolism (e.g., secondary uBAs and secondary metabolites) showed a non-significant reduction (Fig. 1A). Total BA levels between cwCF vs controls showed no statistically significant difference in this analysis (Fig. 1B)—contrary to previous findings in adults with CF (20, 21, 23–25)—although there was a trend toward higher total BA level in cwCF (Fig. 1B), which does parallel previous studies. CF-associated changes in fecal BA pool composition, more so than in overall levels (Fig. 1C, Table S1; Fig. S2A and B), suggest that aberrant BA metabolism begins manifesting early in cwCF. Furthermore, we used a linear model for each of the individual BAs to determine significant differences in log2 transformed concentrations (Fig. S3 through S8; Table S2), accounting for genotype (CF, nonCF) and batch of samples analyzed (batch 1 or batch 2). Of the 84 BAs detected, only 7 alpha OH 3 oxo 4 cholestenoic acid has an adjusted *P*-value < 0.05 (Table S2) and thus a statistically significant difference in the CF vs nonCF cohort.

We identified the BA species most differentially abundant between cwCF and non-CF controls by calculating the difference in mean log2 concentrations for each bile acid between genotypes. To visualize these shifts, we plotted the log2-transformed concentrations of each bile acid species from cwCF and non-CF stool samples against their respective genotype averages, highlighting the BA species with the greatest differences (Fig. S2A). Using this analysis, the top differentially abundant BA species for both genotypes were selected to develop a focused BA panel for subsequent studies (Table S3).

**Fig 2:**
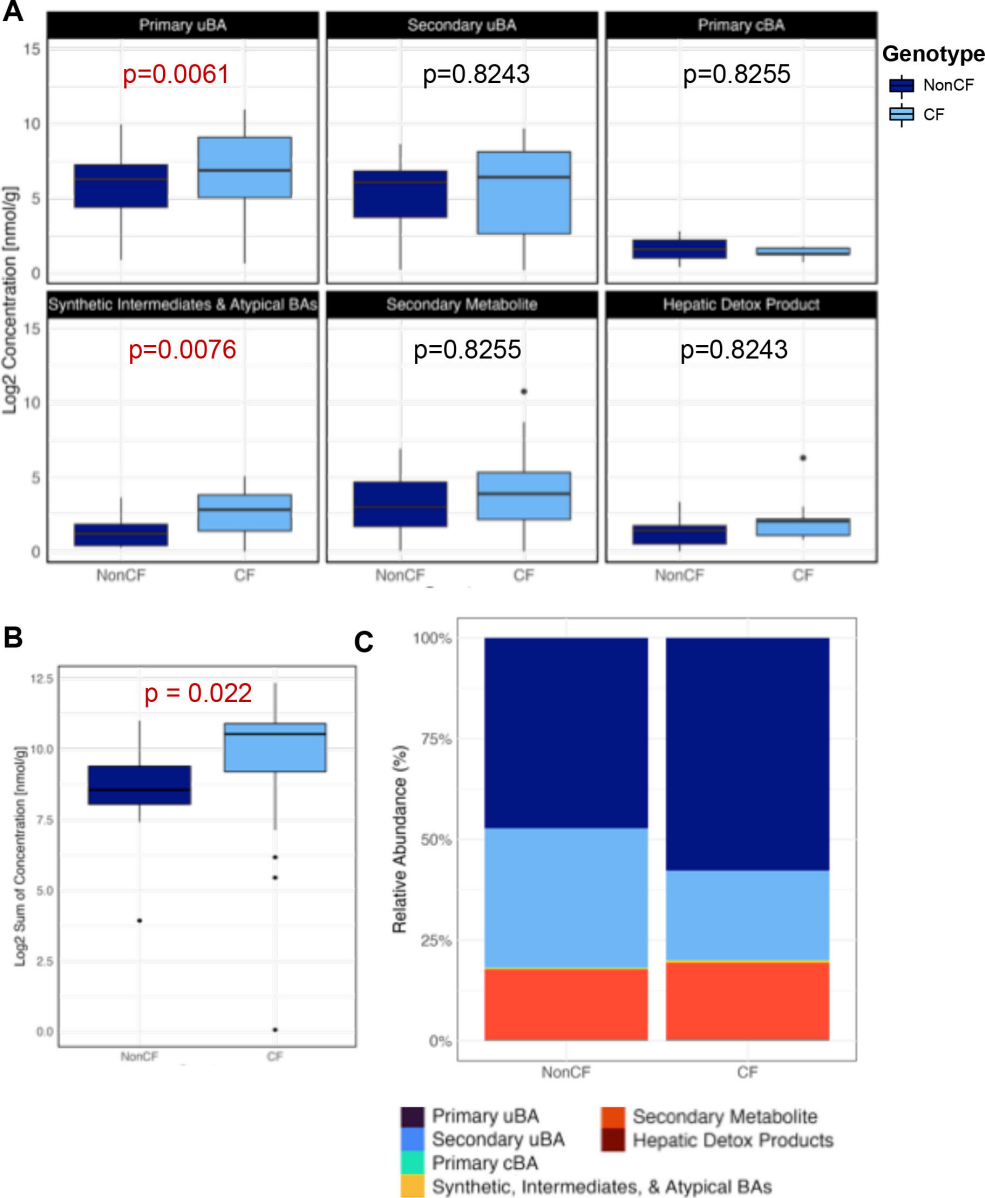
Summary of profiles from focused panel of BA from human stool samples. (**A**) Box plots comparing log2 transformed concentration for each of the six functional groups from human fecal samples (*n* = 18) for each genotype (CF and nonCF). Data analysis performed by linear model, which is summarized in Tables S4 and S5. *P*-values included, with those <0.05 indicated in red. (**B**) Total bile acid concentration (log2 transformed) for each sample from the same human fecal samples in Panel A (*n* = 18) of each genotype (CF and nonCF). A Wilcoxon rank-sum test, consistent with methodologies previously focused on adult populations (20), revealed a significant difference between the sums of BA excretion between genotypes (*P* = 0.022). P-value included and *P* < 0.05 indicated in red. (**C**) Relative abundance of functional groups between genotypes (CF and nonCF) in human samples.

To explore BA species further, we next analyzed a focused panel of BA consisting of the 21 most differentially abundant BA species from our comprehensive analysis in Fig. 1, including known bacterial metabolites (listed in Table S3). The focused BA panel was measured in 18 CF and 18 nonCF stools, which included the 30 individual fecal samples analyzed in Fig. 1 plus 3 additional samples for each genotype. The number 18 was chosen based on our power analysis. In all cases, we used one stool sample per individual. Comparison of log2 transformed concentration of BA classes showed a significant increase for two of the classes in cwCF: primary uBA as well as synthetic, intermediates, and atypical BAs (Fig. 2A, Table S4). Analysis of the focused BA panel shows a significant increase in BA levels in CF samples compared to healthy controls (Fig. 2B), as has been seen in adult populations (20). We noted modest differences in classes of BA between CF and non-CF samples (Fig. 2C, Fig. S2C). Among individual BA species, linear modeling with corrections for multiple comparisons indicates that only beta muricholic acid and allocholic acid are different between groups after adjusting for multiple comparisons (Fig. S9 and S10; Table S5). Enrichment of unconjugated primary BAs, together with the overall increase in fecal BA level, is consistent with both BA and fat malabsorption noted in CF.

We also plotted the primary uBA vs the primary conjugated BA for each CF and nonCF subject (Fig. S2D). This analysis revealed that increases in fecal primary uBAs among cwCF correlated with commensurate elevations in primary cBA precursors, suggesting that CF-related increases in fecal BA levels generally, and fecal primary uBAs specifically, at least partly involves a combination of elevated hepatic BA synthesis and impaired ileal BA absorption.

### Metagenomic analysis of human fecal samples

To explore the microbial composition associated with the human samples, we performed shotgun metagenomic sequencing in 15 CF and 15 nonCF human fecal samples, for which we had parallel comprehensive BA profiles (see Fig. 1). α-Diversity was calculated using the Shannon Diversity Index, accounting for both richness (number of species) and evenness (distribution of species abundances). Overall, no significant differences were found between nonCF and CF samples (Fig. S11) although there was a trend toward lower α-diversity for CF samples, which is expected based on previous findings and perhaps consistent with a reduced effect sizes in young children.

Taxonomic abundances indicate sample CF4 is a clear outlier, showing that 80% of total abundance is dominated by only two phyla (Fig. S12), while CF4, CF8, and CF14 show particularly high abundances of Proteobacteria (Fig. S12). A trend in the relative abundance of *Bacteroides* (reduced) was noted (Fig. S13).

**Fig 3:**
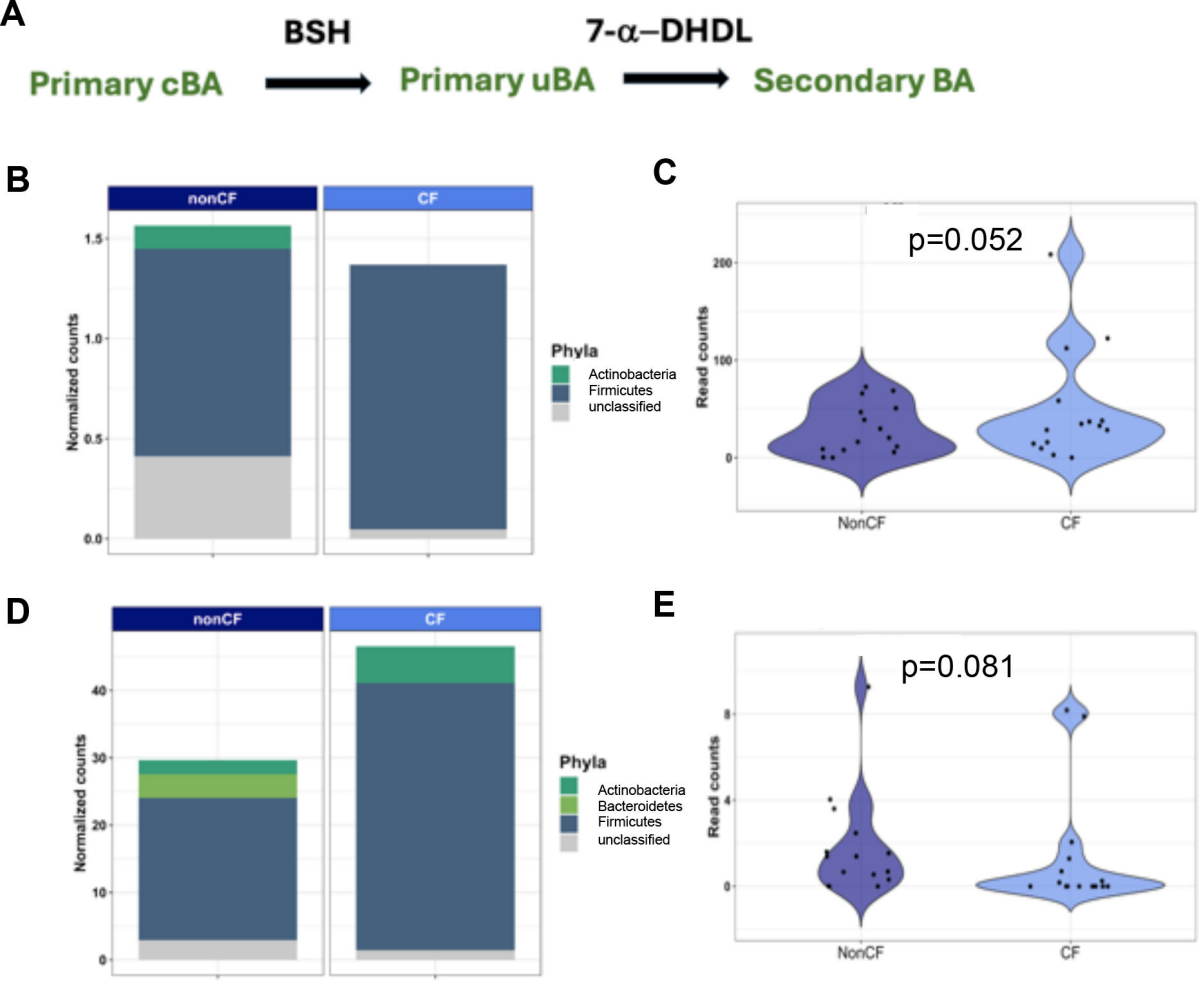
Analysis of microbial bile metabolism genes in human stool samples. (**A**) Simplified bacterial BA catabolism pathway showing the role of BSH in the deconjugation of primary BA, and 7-α-dehydroxylase participating in the conversion of these molecules to secondary BA. (**B**) Normalized counts of the *bsh* gene in CF and nonCF controls. Reads were mapped to the UniRef_90 database, which represents gene families clustered at 90% identity. Clustering at 90% identity enables assignment of taxonomic groups for most gene families. Counts for each gene family are first normalized for gene length with reads per kilobase and then normalized for library depth with total sum scaling to enable comparison between samples. Read counts for each gene families were averaged across samples from the same genotype (nonCF and CF), and the sum of the average read count for gene families representing bile salt hydrolase is represented for each genotype. (**C**) The read counts for all *bsh* gene families were summed for nonCF and CF samples and the distributions tested for significant differences between groups using the Wilcox test (*P* = 0.052). *P*-value included. (**D**) Normalized counts of the *baiE* gene, which codes for the 7-a-dehydroxylase. These data were generated as described in panel **B**. (**E**) The read counts for all gene families encoding 7-α-dehydroxylases were summed for nonCF and CF samples and the distributions tested for significant different between groups using the Wilcox test (*P* = 0.081). *P* value included.

We next mined our metagenomes for abundance of BA metabolism-related genes (Fig. 3A). We observed a trend toward reduced levels of *bsh* gene families within stool microbial communities of cwCF (Fig. 3B and C), whereas a key member of the *bai* operon, *baiE,* which encodes 7-α-dehydroxylase and acts downstream of *bsh*, shows a modest and non-significant increase in the CF samples (Fig. 3D and E). Together, these data suggest that the depletion of bacterially-derived secondary BA species and metabolites in cwCF (Fig. 2) may be attributed to lower rates of the first step in bacterial BA biotransformation, deconjugation via *bsh*. At the same time, CF-related increases in primary uBA species—that are direct products of *bsh*-mediated deconjugation and that parallel increases in primary cBA precursors (Fig. S2D)—could be even higher in cwCF if not for dysbiosis limiting community-wide BA deconjugation machinery.

We next asked if CF-related shifts in BA composition are due to dysbiosis of specific microbes. Consistent with this hypothesis, circos plots indicating correlations between select BA species and phyla show clear differences in the association between phyla and BA for nonCF vs CF samples (Fig. S14A and B). For example, for CF samples, there is a negative correlation between Bacteroidetes and the primary cBA, cholic acid, as would be expected given that this group of organisms can carry the *bsh* genes. Interestingly, for CF samples, many of the correlations between BA are with low abundance phyla that do not change significantly between CF and nonCF samples (Fig. S14C), indicating perhaps a complex relationship between microbial community composition and BA abundance that may be additionally influenced by changes in host intestinal BA absorption.

### Characterizing mouse and ferret BA profiles to assess their utility for studying BA in CF

**Fig 4:**
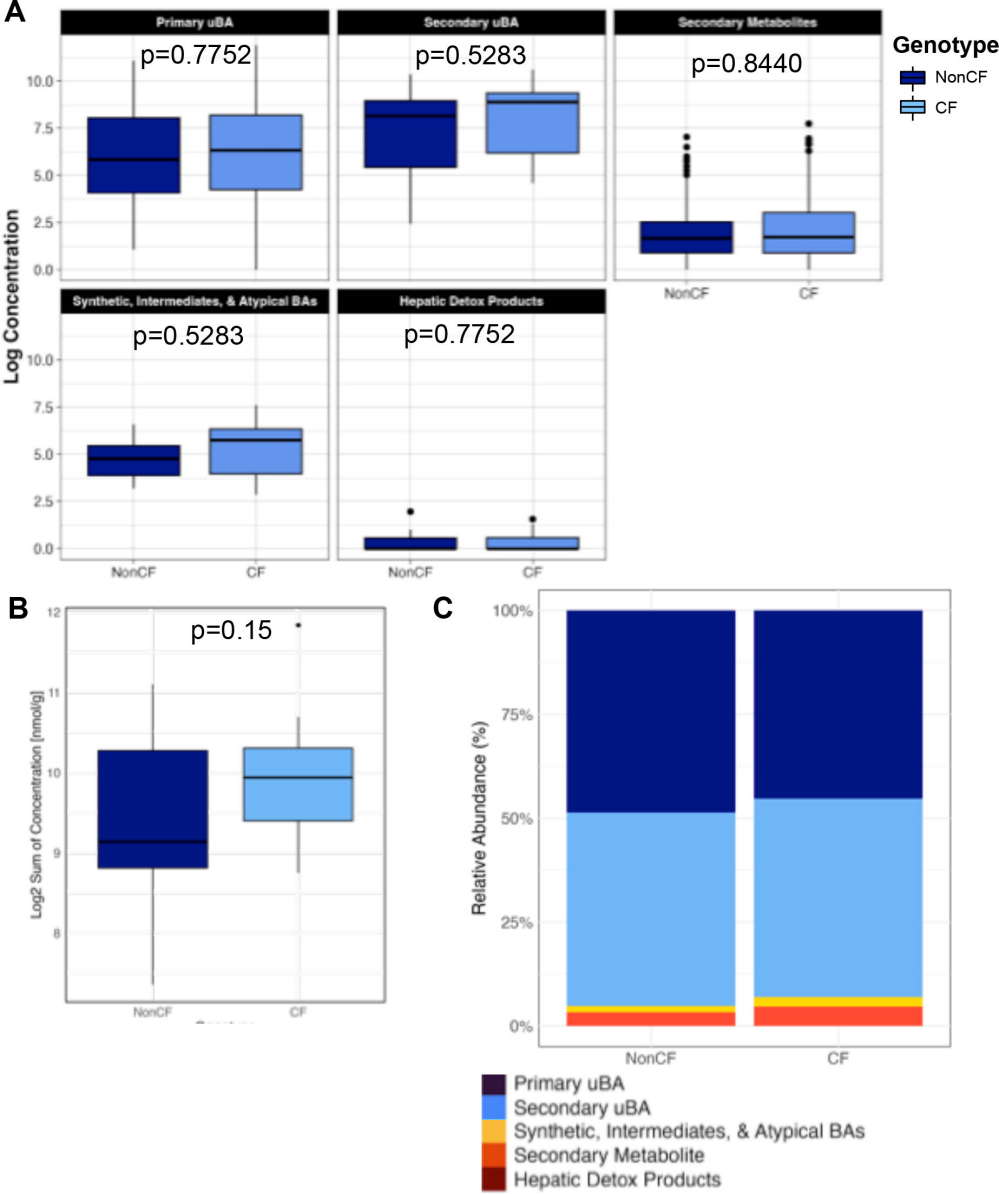
Summary of focused BA profiles from mouse stool samples. (**A**) Box plots comparing log2 transformed concentration for each functional group from mouse fecal samples (*n* = 15) of each genotype (CF and nonCF). Data analysis performed by linear model, which is summarized in Tables S6 and S7. *P*-values are shown. (**B**) Total bile acid concentration (log transformed) for each genotype from the same mouse fecal samples in Panel A (*n* = 15) of each genotype (CF and nonCF). Linear model does not show significance (*P*-value = 0.15). (**C**) Relative abundance of functional groups between genotypes (CF & nonCF) in mouse samples.

To determine whether or how CF-related changes in BA metabolism are reflected in common animal models, we used the same focused BA panel (Table S3) to analyze fecal BAs in mouse and ferret models of CF, compared with wild-type controls. Data from WT C57BL/6 mice (nonCF) and C57BL/6 *cftr*F508del (CF) showed no significant differences in either total BA levels or levels of select BA classes between CF and nonCF animals although CF mice again showed trends toward increased fecal levels of both primary uBAs and synthetic intermediates, atypical BAs (Fig. 4, Fig. S15 through S17; Table S6 and S7). Linear modeling with corrections for multiple comparisons indicates that dehydrolithocholic acid is the only individual BA species from the mouse BA pool that is significantly different in CF vs control mice (Table S7).

**Fig 5:**
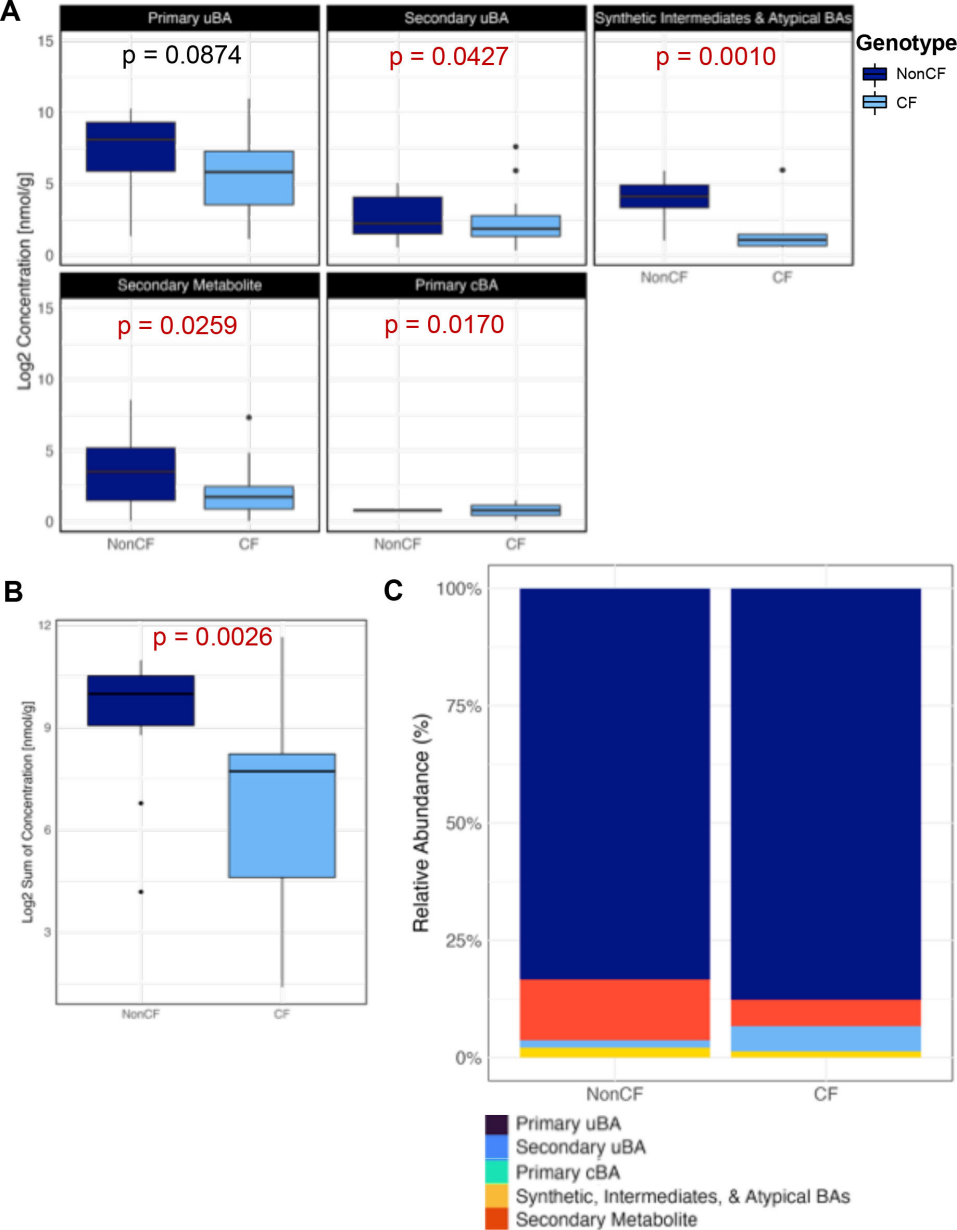
Summary of focused BA profiles from ferret stool samples. (**A**) Box plots comparing log2 transformed concentration for each functional group from ferret fecal samples (*n* = 15) of each genotype (CF and nonCF). Data analysis performed by linear model, which is summarized in Tables S8 and S9. *P*-values included, with those <0.05 indicated in red. (**B**) Total bile acid concentration (log2 transformed) for each genotype from the same ferret fecal samples in Panel A (*n* = 15) of each genotype (CF and nonCF). The linear model shows significance (*P*-value = 0.0026), indicated in red since *P* < 0.05. (**C**) Relative abundance of functional groups between genotypes (CF and nonCF) in ferret samples.

Ferret samples show more statistically significant differences in CF-related fecal BA profiles although several of these changes are paradoxical relative to our human findings. CF ferrets do show a modest but significant increase in fecal levels of primary cBA, akin to cwCF, while all other BA classes—and total fecal BA levels—are reduced compared with wild-type controls (Fig. 5A through C, Fig. S18 through S20; Table S8). Furthermore, measuring total BA levels shows that nonCF ferrets have significantly more BAs in their stool than ferrets carrying the mutation causing CF (Fig. 5B), in contrast to the literature (20, 25, 27) and our human data presented here showing more BA in CF (Fig. 2B). When investigating individual BA species, linear modeling with multiple corrections indicates 9/23 BA are significantly different in CF (Fig. S18 through S20; Table S9). These data show clear differences between human data and data collected from the ferret model.

**Fig 6:**
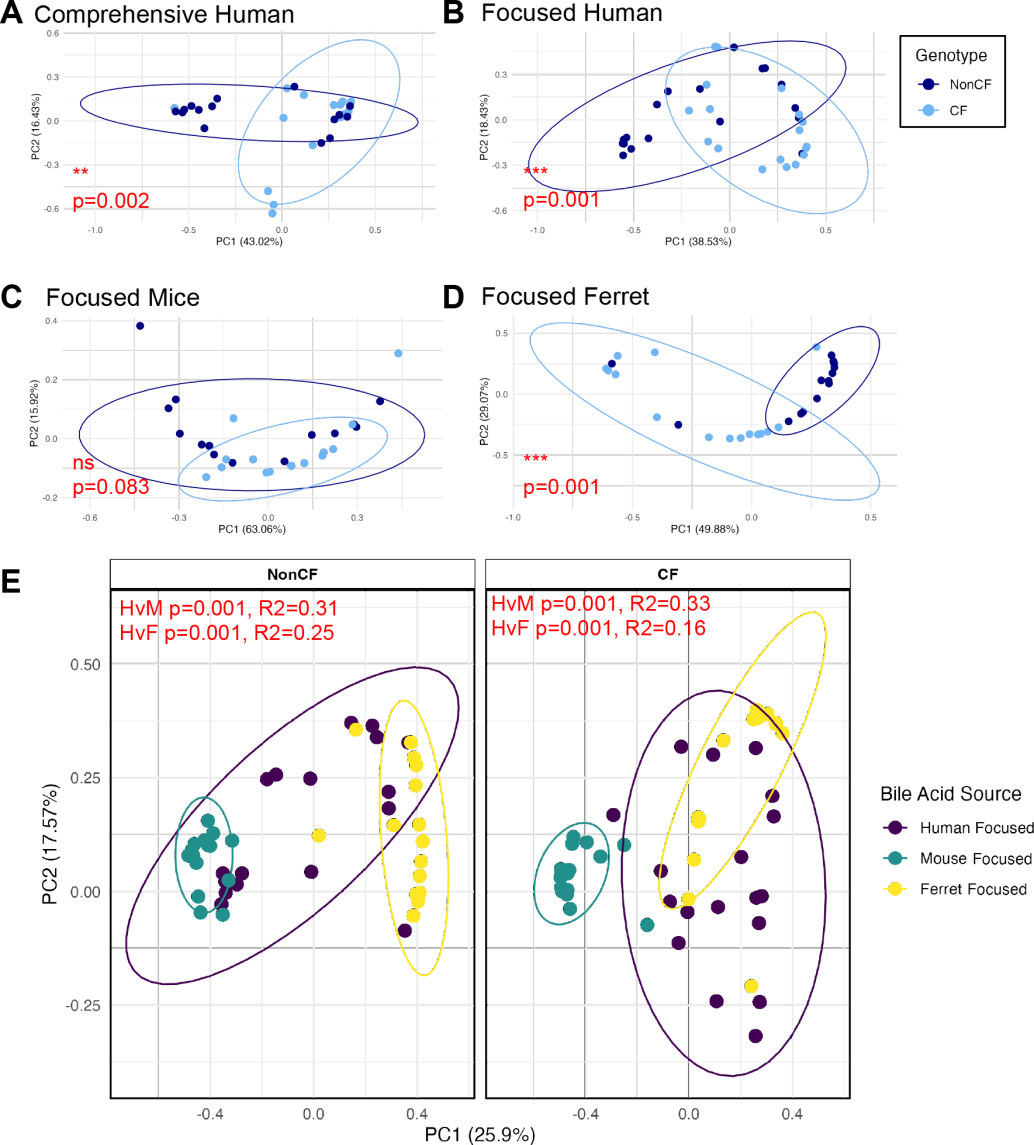
Bray Curtis beta analysis between nonCF and CF for (**A**) comprehensive panel of human bile acids (*n* = 89 BA), (**B**) focused panel of human bile acids (*n* = 21), (**C**) focused panel of mouse bile acids, and (**D**) focused panel of ferret bile acids. (**E**) Bray Curtis beta analysis between bile acids sample sources for nonCF (left) and CF (right) samples. Statistical analysis was performed by PERMANOVA, where *P* and *R*-squared values are indicated in red. Pairwise-PERMANOVA was performed for data in panel E, where *P*-values indicating significance of human vs mouse are indicated by HvM, and human vs ferret indicated by HvsF.

The observation that we saw more differences in the BA between CF and nonCF ferrets prompted us to examine the BA profiles in more detail. Analysis of the ferret BA pools showed a significant difference in the β-diversity of BA for the CF vs nonCF populations, which was analogous to what was observed for the human cohort (Fig. 6A, B and D). There is no significant difference in the β-diversity of BA for the mouse CF and nonCF samples (Fig. 6C). Fig. 6E shows an overlap in BA profiles for nonCF samples from humans, mice, and ferrets, but there is little overlap specifically between mice and ferrets. An overlap in BA profiles for CF samples is only observed for persons and ferrets with CF. In all cases, the BA profiles are significantly different between humans and animal models.

## DISCUSSION

Our findings provide a comprehensive analysis of BA profiles in fecal samples from cwCF compared to healthy controls, highlighting unique aspects of BA levels in CF. Total BA levels were significantly higher in the cwCF samples, and distinct differences in BA composition were observed. The increase in primary uBAs underscores the potential effects of microbial dysbiosis impairing BA metabolism in pwCF, either alone or in combination with changes in host intestinal BA absorption, while differences in biosynthetic intermediates and atypical BAs for CF samples likely reflect differences in hepatic metabolism. We note that we do not observe significant differences in total fecal BA levels when analyzing the large, comprehensive panel of 89 BA, whereas we do see such differences for the focused BA analysis (*n* = 21 BA). The latter observation has been what was reported previously, as described above for both CF and inflammatory bowel disease.

Our metagenomic analysis provides data that can be mined to assess questions related to microbial BA metabolism because we can link microbial communities and genes directly to the level of BA measured given these data were acquired from the same samples. In this small cohort, we did not observe statistical significance in BA-metabolic genes but trends toward a decrease in *bsh* abundance and a modest increase in the gene coding for 7-α-dehydroxylase (*baiE*) in CF were observed. Overall, these data also provide a resource for investigators to start a more detailed analysis of the interactions between BA, the microbiota, and the host. It will be important for future studies to also incorporate meta-transcriptomics analyses, to assess relationships between BAs and not only bacterial BA metabolism-related gene abundance but also their potential expression.

We also investigated the potential use of animal models to provide critical insights into BA metabolism in CF. Mouse models exhibit minimal differences between CF and nonCF genotypes, with only a single BA showing significantly different levels in CF vs nonCF samples. We also noted no changes in bulk levels of BA, which is at odds with the data from cwCF. It is well established that mouse livers, unlike those in humans, utilize a cytochrome P450 enzyme, Cyp2c70, to metabolize the major human primary BA, CDCA, into rodent-biased muricholates (e.g.*,* αMCA, βMCA) (30, 31). MCA species are vastly more hydrophilic, and thus less potentially toxic, compared with CDCA. Hence, the utility of *cftrF508del* mice in the study of CF-related BA functions using stool sampling is unclear and should be approached with caution.

Our data show that the ferret models also display differences from humans. Ferrets show a trend toward reduction in unconjugated primary BA, while humans show a significant increase. Ferrets also show a significant reduction in secondary unconjugated BA, while humans show an increasing trend. Importantly, while pwCF show a significant increase in total BA, CF ferrets show a significant decrease. Interestingly, unlike mice, ferrets show a shift in BA profiles in CF vs nonCF cohorts. However, given the difference with adults and cwCF, care must be taken when using stool samples with this model.

Beyond this work, specific implications for the distinctive bile acid pools in humans, mice, and ferrets—as they relate to CF-associated BA functional studies—include the dearth of key CDCA- and microbe-derived immunoregulatory BAs, namely, LCA and its metabolites, in mice (32–34). There is also the near absence of all secondary BAs (e.g., DCA and LCA) in ferrets that at least partly relate to the evolution of host BA metabolism in mammals (35). Thus, care must be taken when extrapolating findings from animal models to humans regarding BA metabolism.

We note several important limitations of our study. First, the cohort size is relatively small, constrained by the cost of the comprehensive BA analysis. Second, we do not control for the diet of the human subjects, thus this limitation could cause increased variance in our data. Notably, samples from cwCF and animal models were collected in a non-fasting state, and diet and timing of food intake could not be controlled. However, we can note that, in general, the cwCF in our cohort were on a CF standard, high fat, high calorie diet. Third, while we examined the BA profiles of cwCF here—an age range not yet assessed in the literature for both BA levels and metagenomic profiling, we profiled BA in adult animals. Given the overall similarities of BA profiles in children, teens, and adults in human cohorts, our data suggest that the use of adult animals in future studies could be relevant. However, as noted above, the utility of these mouse and ferret models using stool samples for the study of BA in CF is unclear. Given the mild nature of the CF allele in our mouse model used here (36, 37), it may be necessary to explore BA functions in mouse settings that express more severe loss-of-function *Cftr* alleles. Fourth, the CF cohort samples span a large age range (13 days to 7.5 years) as this study has been ongoing for over a decade (7–9, 38–40). Our control cohort is more limited in age (<3 years of age) and from a single daycare center in New Hampshire. We should also note that the CF cohort is also from a single CF center in New Hampshire. Fifth, the CF cohort samples are typically stored in a home freezer for up to several days, which could impact the suite of BA identified, compared to the nonCF cohort, wherein the stool samples were stored in a cooler for a few hours before storage at −80°C. Sixth, we normalized BA levels to g of stool—such values could be impacted by diet, degree of malabsorption, or other differences between the CF and nonCF cohorts causing alterations in stool mass. Finally, here we simply note the stool BA levels and cannot speak to the mechanism whereby the BA levels have shifted.

This study provides a foundation for further exploration of the role of BA metabolism and microbial dysbiosis in CF and provides baseline data for establishing *in vitro* models. Future research should focus on understanding the mechanisms underlying these changes, their impact on disease progression, and the potential for targeted therapeutic interventions to restore BA homeostasis in CF.

## MATERIALS AND METHODS

### CF human cohort sample collection

A total of 15 CF samples were analyzed for the comprehensive, targeted BA panel (*n* = 89 compounds, which we refer to as the “comprehensive” panel here) and metagenomics, one sample per cwCF. These samples were collected as part of the ongoing Dartmouth CF Infant and Children’s Cohort. Eighteen CF samples were analyzed for the focused BA panel—these samples included the same 15 samples used for the large BA panel and metagenomic study plus an additional 3 CF samples from the Dartmouth CF Infant and Children’s Cohort. Only one sample per subject was analyzed. CF fecal samples were collected by parents and initially stored in a home freezer prior to the transfer to the clinic during routine visits with clinicians. At the clinic, the samples were stored at −80°C until they were transported to the lab to be aliquoted, stored in −80°C, and later processed for metabolomics and sequencing. This study was approved by the Dartmouth College Committee for the Protection of Human Subjects (CPHS Study # 00021761).

### NonCF human control cohort collection

Fecal samples were collected at a local daycare center and stored briefly on ice in a cooler prior to the transfer to the lab at Dartmouth. A total of 15 nonCF samples were analyzed for the comprehensive BA panel (*n* = 89 BA) and metagenomics, one sample per individual. A total of 18 nonCF samples were analyzed for the focused BA panel—these samples included the same 15 samples used for the large BA panel and metagenomic study plus an additional three nonCF samples from three additional nonCF individuals. Only one sample per subject was analyzed. At the lab, the samples were aliquoted and stored in −80°C and later processed for metabolomics and sequencing. These are de-identified samples from children <3 years of age. No other additional metadata were collected. This study was approved by the Dartmouth College Committee for the Protection of Human Subjects (CPHS Study #02001623).

### CF and NonCF mouse stool collection

CF mouse fecal samples were collected from 7 female and 8 male Cftr^em1Cwr^ (an F508del with a milder CF phenotype) mice at 9 weeks; we have used these animals in our previous studies (28). NonCF mouse fecal samples were collected from 7 male and 8 female WT C57BL/6 mice at 7 weeks. Studies requiring mice for collection of stool samples were performed in accordance with a protocol that adhered to the *Guide for the Care and Use of Laboratory Animals* of the National Institutes of Health (NIH) and under the supervision of the Institutional Animal Care and Use Committee at Dartmouth College (approval #00002184). The Dartmouth College animal program is registered with the U.S. Department of Agriculture through certificate number 12R-0001, operates in accordance with Animal Welfare Assurance (NIH/PHS) number D16-00166 (A3259-01). The program is accredited with the Association for Assessment and Accreditation of Laboratory Animal Care International (accreditation #398). Age-matched and sex-matched animals were used. Note that these animals were not fasting before collection of samples.

### CF and NonCF ferret stool collection

Ferret fecal samples were provided by J. Engelhardt at the University of Iowa. Ferrets were raised as described previously (41). Samples were collected, aliquoted, and stored in −80°C prior to shipping to Dartmouth. CF ferret samples included 8 females and 7 males, ages ranging from 6 to 30 months. CF ferrets were off ivacaftor (VX-770), a medication used to treat CF, for an average of 133 days. Ivacaftor targets the underlying protein defect in CF and enhances ion transport across epithelial cell membranes, thereby alleviating the buildup of thick, viscous mucus (42). NonCF ferret samples included 6 females and 9 males, ages ranging from 6 to 34 months. These studies were approved by the University of Iowa Institutional Animal Care and Use Committee. The list of animals used in this study and associated metadata is shown in Table S10. Note that there was no fasting before collection of samples.

### Comprehensive metabolite quantification and analysis (creative proteomics)

Stool samples were stored frozen at −80 degrees prior to metabolite quantification. Thirty human fecal samples (100 mg/sample; 15 CF and 15 nonCF) were shipped frozen to Creative Proteomics for bile acid quantification by Ultra Performance Liquid Chromatography Mass Spectrometry (UPLC-MS/MS). Samples were lyophilized to dryness, weighed, and added to a 2 mL homogenizing tube. The samples were homogenized in 20 µL of a 75% acetonitrile solution per mg of biomass on a MM 400 mill mixer, at 30 Hz for 3 min, followed by sonication in an ice-water bath for 5 min. Samples were centrifuged to remove debris. Clear supernatant of each sample was diluted 10-fold with internal standard (IS) solution. Five microliter aliquots of the resultant sample solutions of each of the calibrated solutions were resolved via UPLC-MS/MS.

An Agilent 1290 UHPLC system coupled to an Agilent 6495 QQQ mass spectrometer was used to analyze the BA profiles. The MS instrument was operated in the multiple reaction monitoring (MRM) mode with negative ion detection. A Waters C18 column (2.1*150 mm, 1.7 µM) was used for LC separation, and the mobile phase was 0.01% formic acid in water and in acetonitrile for binary—solvent gradient elution. A mixture of standard substances of all bile acids at 10 µM for each compound was prepared in an IS solution of 14 deuterium-labeled bile acids. This solution was further diluted step by step to have 9-point calibration solutions.

Concentrations of detected bile acids were calculated by interpolating the internal standard calibrated linear-regression curves of individual bile acids, with the analyte-to-IS peak area ratios measured from injections of the sample solutions. The full data set is shown in Table S11.

### Focused metabolite quantification and analysis (Michigan Core)

A focused subset of BA (Table S3) was performed using LC-MS. Stool samples were stored frozen at −80°C prior to metabolite quantification. Thirty-six human fecal samples (18/per genotype) were thawed on ice for 30 min. Stool was weighed (<10 mg) in an Eppendorf tube, and 1 mL of extraction buffer (0.2 g butylated hydroxytoluene in 2 mL milliQ-water) was added to the stool sample. Samples were vortexed for 1 min, placed on ice, centrifuged at 10,000 *g* at 4°C for 10 min. Following centrifugation, 750 µl of the supernatant was transferred to a 2 mL glass vial to be shipped to Michigan State University Mass Spectrometry and Metabolomics Core for analysis. The full data set is shown in Table S12.

### Description of statistical analysis for BA

Concentrations of BA are normalized by stool weight then log_2_ transformed. Any BA that was below the limit of detection in all samples (both CF and nonCF) were removed from further analysis. When samples had a concentration of 0 in a specific bile acid, a value of 1 was added to the last decimal place so that normalization (log2 transformation) across all samples could be conducted. In the comprehensive BA profiling, we could detect 84 out of 89 BAs in at least one sample. Those left out were (Chenodeoxycholic acid 3G, Lithocholic acid 24G, Hyodeoxycholic acid 3 glucuronide, Hyodeoxycholic acid 24 glucuronide, and Ursodeoxycholic acid 3 glucuronide). In order to determine statistical differences by genotype (nonCF or CF) on bile acid concentrations, we ran simple and mixed effects linear models on each individual bile acid and each bile acid functional group, respectively. We set nonCF as the reference, and set log2 transformed concentrations of BAs as the independent variable, accounting for genotype and batch (1 or 2) as the dependent variables. For bile acid functional group, we also take into account repeated measures from samples by setting sample (or human participant, mouse, or ferret) as a random effect. *P*-values were then adjusted for multiple comparisons (84) by False Discovery Rate (FDR) at a threshold of 0.05. To assess differences in bile acid composition between sample sources, we performed Principal Coordinates Analysis (PCoA). Briefly, we calculated a Bray-Curtis β-diversity dissimilarity matrix and visualized it using PCoA. To assess statistical significance, we ran pairwise PERMANOVA tests within each genotype comparing human vs mouse samples and human vs ferret samples. To evaluate the relationship between primary conjugated bile acids (cBA) and primary unconjugated bile acids (uBA), bile acid concentrations were summed per sample by functional group for each metabolomics method (comprehensive and focused) in human samples. Statistical analyses, including Spearman correlation tests, were conducted to assess associations between primary cBA and primary uBA concentrations by genotype and metabolomics method. The coefficient of determination (*R*²) and *P*-values were reported to evaluate the strength and significance of these relationships. Data processing, visualization, and statistical analysis were performed in *R* (v4.4.2) using the packages readr for data import, dplyr and tidyr for data manipulation, ggplot2 for plotting, lme4 and lmerTest for linear mixed-effects modeling, and vegan for diversity analyses and PERMANOVA testing. The code is available at https://github.com/GeiselBiofilm.

### Shotgun metagenomic sequencing

DNA for metagenomics analysis was quantified by Qubit (Thermo Fisher). One hundred fifty nanograms of DNA was used as input into the Illumina DNA Prep kit (Illumina) for library preparation following manufacturer’s instructions. Libraries were pooled for sequencing on a NextSeq2000 instrument targeting 30M, paired-end 150 bp reads per sample. The data are available under BioProject PRJNA1244851.

### Metagenomic analysis

To assess microbial diversity metagenomic data were analyzed using the standard metagenomics workflow used by the Dartmouth Genomic Data Science Core. Briefly, this workflow involves filtering host reads using KneadData v. 0.12.0, taxonomic assignment using metaphlan v. 4.0.6 with the mpa_vOct22_CHOCOPhlAnSGB_202212 database, gene family and pathway assignment with humann v. 3.7 with the full_chocophlan.v201901_v31 and uniref90_annotated_v201901b_full databases, and lastly differential abundance testing across conditions of interest with MaAslin v. 1.14.1 (43–46). All code is available upon request.

## SUPPLEMENTAL MATERIAL

Supplemental Figures spectrum.01451-25-s0001.pdf Figures S1 to S20.

Supplemental Tables spectrum.01451-25-s0002.pdf Tables S1 to S9.

Table S10 spectrum.01451-25-s0003.xlsx Information regarding ferret stool samples used in this study.

Table S11 spectrum.01451-25-s0004.xlsx Creative Proteomics comprehensive panel.

Table S12 spectrum.01451-25-s0005.xlsx Focused bile panel.

ASM does not own the copyrights to Supplemental Material that may be linked to, or accessed through, an article. The authors have granted ASM a non-exclusive, world-wide license to publish the Supplemental Material files. Please contact the corresponding author directly for reuse.

## References

[1] De Lisle RC, Borowitz D. 2013. The cystic fibrosis intestine. Cold Spring Harb Perspect Med 3:a009753. doi:10.1101/cshperspect.a009753 23788646 PMC3753720

[2] Meeker SM, Mears KS, Sangwan N, Brittnacher MJ, Weiss EJ, Treuting PM, Tolley N, Pope CE, Hager KR, Vo AT, Paik J, Frevert CW, Hayden HS, Hoffman LR, Miller SI, Hajjar AM. 2020. CFTR dysregulation drives active selection of the gut microbiome. PLoS Pathog 16:e1008251. doi:10.1371/journal.ppat.1008251 31961914 PMC6994172

[3] Price CE, O’Toole GA. 2021. The gut-lung axis in cystic fibrosis. J Bacteriol 203:e0031121. doi:10.1128/JB.00311-21 34339302 PMC8459759

[4] de Freitas MB, Moreira EAM, Tomio C, Moreno YMF, Daltoe FP, Barbosa E, Ludwig Neto N, Buccigrossi V, Guarino A. 2018. Altered intestinal microbiota composition, antibiotic therapy and intestinal inflammation in children and adolescents with cystic fibrosis. PLoS One 13:e0198457. doi:10.1371/journal.pone.0198457 29933382 PMC6014676

[5] Garg M, Ooi CY. 2017. The enigmatic gut in cystic fibrosis: linking inflammation, dysbiosis, and the increased risk of malignancy. Curr Gastroenterol Rep 19:6. doi:10.1007/s11894-017-0546-0 28155088

[6] Lazzarotto ES, Vasco JF de M, Führ F, Riedi CA, Filho NAR. 2023. Systematic review on fecal calprotectin in cystic fibrosis. J Pediatr (Rio J) 99:4–10. doi:10.1016/j.jped.2022.01.006 35523321 PMC9875247

[7] Antosca KM, Chernikova DA, Price CE, Ruoff KL, Li K, Guill MF, Sontag NR, Morrison HG, Hao S, Drumm ML, MacKenzie TA, Dorman DB, Feenan LM, Williams MA, Dessaint J, Yuan IH, Aldrich BJ, Moulton LA, Ting L, Martinez-del Campo A, Stewart EJ, Karagas MR, O’Toole GA, Madan JC. 2019. Altered stool microbiota of infants with cystic fibrosis shows a reduction in genera associated with immune programming from birth. J Bacteriol 201:e00274–19. doi:10.1128/JB.00274-19 31209076 PMC6657602

[8] Price CE, Hampton TH, Valls RA, Barrack KE, O’Toole GA, Madan JC, Coker MO. 2023. Development of the intestinal microbiome in cystic fibrosis in early life. mSphere 8:e0004623. doi:10.1128/msphere.00046-23 37404016 PMC10449510

[9] Verster AJ, Salerno P, Valls R, Barrack K, Price CE, McClure EA, Madan JC, O’Toole GA, Sanville JL, Ross BD. 2025. Persistent delay in maturation of the developing gut microbiota in infants with cystic fibrosis. mBio 16:e0342024. doi:10.1128/mbio.03420-24 39945545 PMC11898760

[10] Hayden HS, Eng A, Pope CE, Brittnacher MJ, Vo AT, Weiss EJ, Hager KR, Martin BD, Leung DH, Heltshe SL, Borenstein E, Miller SI, Hoffman LR. 2020. Fecal dysbiosis in infants with cystic fibrosis is associated with early linear growth failure. Nat Med 26:215–221. doi:10.1038/s41591-019-0714-x 31959989 PMC7018602

[11] Manor O, Levy R, Pope CE, Hayden HS, Brittnacher MJ, Carr R, Radey MC, Hager KR, Heltshe SL, Ramsey BW, Miller SI, Hoffman LR, Borenstein E. 2016. Metagenomic evidence for taxonomic dysbiosis and functional imbalance in the gastrointestinal tracts of children with cystic fibrosis. Sci Rep 6:22493. doi:10.1038/srep22493 26940651 PMC4778032

[12] Burke DG, Fouhy F, Harrison MJ, Rea MC, Cotter PD, O’Sullivan O, Stanton C, Hill C, Shanahan F, Plant BJ, Ross RP. 2017. The altered gut microbiota in adults with cystic fibrosis. BMC Microbiol 17:58. doi:10.1186/s12866-017-0968-8 28279152 PMC5345154

[13] Thavamani A, Salem I, Sferra TJ, Sankararaman S. 2021. Impact of altered gut microbiota and its metabolites in cystic fibrosis. Metabolites 11:123. doi:10.3390/metabo11020123 33671639 PMC7926988

[14] Barrack KE, Hampton TH, Valls RA, Surve SV, Gardner TB, Sanville JL, Madan JL, O’Toole GA. 2024. An in vitro medium for modeling gut dysbiosis associated with cystic fibrosis. J Bacteriol 206:e0028623. doi:10.1128/jb.00286-23 38169295 PMC10810206

[15] Werlin SL, Benuri‐Silbiger I, Kerem E, Adler SN, Goldin E, Zimmerman J, Malka N, Cohen L, Armoni S, Yatzkan‐Israelit Y, Bergwerk A, Aviram M, Bentur L, Mussaffi H, Bjarnasson I, Wilschanski M. 2010. Evidence of intestinal inflammation in patients with cystic fibrosis. J pediatr gastroenterol nutr 51:304–308. doi:10.1097/MPG.0b013e3181d1b013 20512061

[16] Figliuolo VR, Dos Santos LM, Abalo A, Nanini H, Santos A, Brittes NM, Bernardazzi C, de Souza HSP, Vieira LQ, Coutinho-Silva R, Coutinho CMLM. 2017. Sulfate-reducing bacteria stimulate gut immune responses and contribute to inflammation in experimental colitis. Life Sci 189:29–38. doi:10.1016/j.lfs.2017.09.014 28912045

[17] Guo FF, Yu TC, Hong J, Fang JY. 2016. Emerging roles of hydrogen sulfide in inflammatory and neoplastic colonic diseases. Front Physiol 7:156. doi:10.3389/fphys.2016.00156 27199771 PMC4853395

[18] Hughes ER, Winter MG, Duerkop BA, Spiga L, Furtado de Carvalho T, Zhu W, Gillis CC, Büttner L, Smoot MP, Behrendt CL, Cherry S, Santos RL, Hooper LV, Winter SE. 2017. Microbial respiration and formate oxidation as metabolic signatures of inflammation-associated dysbiosis. Cell Host & Microbe 21:208–219. doi:10.1016/j.chom.2017.01.005 28182951 PMC5313043

[19] Guzior DV, Quinn RA. 2021. Review: microbial transformations of human bile acids. Microbiome 9:140. doi:10.1186/s40168-021-01101-1 34127070 PMC8204491

[20] O’Brien S, Mulcahy H, Fenlon H, O’Broin A, Casey M, Burke A, FitzGerald MX, Hegarty JE. 1993. Intestinal bile acid malabsorption in cystic fibrosis. Gut 34:1137–1141. doi:10.1136/gut.34.8.1137 8174969 PMC1374370

[21] van de Peppel IP, Bodewes FAJA, Verkade HJ, Jonker JW. 2019. Bile acid homeostasis in gastrointestinal and metabolic complications of cystic fibrosis. J Cyst Fibros 18:313–320. doi:10.1016/j.jcf.2018.08.009 30201330

[22] Urdaneta V, Casadesús J. 2017. Interactions between bacteria and bile salts in the gastrointestinal and hepatobiliary tracts. Front Med 4:163. doi:10.3389/fmed.2017.00163 PMC5632352 29043249

[23] Bodewes FAJA, van der Wulp MYM, Beharry S, Doktorova M, Havinga R, Boverhof R, James Phillips M, Durie PR, Verkade HJ. 2015. Altered intestinal bile salt biotransformation in a cystic fibrosis (Cftr−/−) mouse model with hepato-biliary pathology. J Cyst Fibros 14:440–446. doi:10.1016/j.jcf.2014.12.010 25633479

[24] Strandvik B, Einarsson K, Lindblad A, Angelin B. 1996. Bile acid kinetics and biliary lipid composition in cystic fibrosis. J Hepatol 25:43–48. doi:10.1016/S0168-8278(96)80326-6 8836900

[25] Weber AM, Roy CC, Chartrand L, Lepage G, Dufour OL, Morin CL, Lasalle R. 1976. Relationship between bile acid malabsorption and pancreatic insufficiency in cystic fibrosis. Gut 17:295–299. doi:10.1136/gut.17.4.295 773791 PMC1411100

[26] Thompson MH. 1985. Fecal bile acids in health and disease. In Galli G, Bosisio E (ed), Liver, Nutrition, and Bile Acids. Springer, Boston, MA.

[27] Jonas A, Diver-Haber A. 1988. Bile acid sequestration by the solid phase of stools in cystic fibrosis patients. Digest Dis Sci 33:724–731. doi:10.1007/BF01540437 3371142

[28] Price C, Valls R, Ramsey A, Loeven N, Jones J, Barrack K, Schwartzman J, Royce D, Cramer R, Madan J, Ross B, Bliska J, O’Toole G. 2024. Intestinal Bacteroides modulates systemic inflammation and the microbial ecology in a mouse model of CF: evidence for propionate and other short chain fatty acids reducing systemic inflammatory cytokines mBio:e03144–23. doi:10.1016/S1569-1993(23)00942-6 38179971 PMC10865972

[29] Sudo K, Delmas-Eliason A, Soucy S, Barrack KE, Liu J, Balasubramanian A, Shu CJ, James MJ, Hegner CL, Dionne HD, Rodriguez-Palacios A, Krause HM, O’Toole GA, Karpen SJ, Dawson PA, Schultz D, Sundrud MS. 2024. Quantifying forms and functions of enterohepatic bile acid pools in mice. Cell Mol Gastroenterol Hepatol 18:101392. doi:10.1016/j.jcmgh.2024.101392 39179177 PMC11490680

[30] Klindt C, Truong JK, Bennett AL, Pachura KJ, Herebian D, Mayatepek E, Luedde T, Ebert M, Karpen SJ, Dawson PA. 2024. Hepatic bile acid accretion correlates with cholestatic liver injury and therapeutic response in Cyp2c70 knockout mice with a humanized bile acid composition . American Journal of Physiology-Gastrointestinal and Liver Physiology 327:G789–G809. doi:10.1152/ajpgi.00129.2024 39350733 PMC11684888

[31] Truong JK, Bennett AL, Klindt C, Donepudi AC, Malla SR, Pachura KJ, Zaufel A, Moustafa T, Dawson PA, Karpen SJ. 2022. Ileal bile acid transporter inhibition in Cyp2c70 KO mice ameliorates cholestatic liver injury. J Lipid Res 63:100261. doi:10.1016/j.jlr.2022.100261 35934110 PMC9460185

[32] Hang S, Paik D, Yao L, Kim E, Trinath J, Lu J, Ha S, Nelson BN, Kelly SP, Wu L, Zheng Y, Longman RS, Rastinejad F, Devlin AS, Krout MR, Fischbach MA, Littman DR, Huh JR. 2019. Bile acid metabolites control TH17 and Treg cell differentiation. Nature 576:143–148. doi:10.1038/s41586-019-1785-z 31776512 PMC6949019

[33] Li W, Hang S, Fang Y, Bae S, Zhang Y, Zhang M, Wang G, McCurry MD, Bae M, Paik D, Franzosa EA, Rastinejad F, Huttenhower C, Yao L, Devlin AS, Huh JR. 2021. A bacterial bile acid metabolite modulates Treg activity through the nuclear hormone receptor NR4A1. Cell Host & Microbe 29:1366–1377. doi:10.1016/j.chom.2021.07.013 34416161 PMC9064000

[34] Song X, Sun X, Oh SF, Wu M, Zhang Y, Zheng W, Geva-Zatorsky N, Jupp R, Mathis D, Benoist C, Kasper DL. 2020. Microbial bile acid metabolites modulate gut RORγ+ regulatory T cell homeostasis. Nature 577:410–415. doi:10.1038/s41586-019-1865-0 31875848 PMC7274525

[35] Hofmann AF, Hagey LR. 2014. Key discoveries in bile acid chemistry and biology and their clinical applications: history of the last eight decades. J Lipid Res 55:1553–1595. doi:10.1194/jlr.R049437 24838141 PMC4109754

[36] Aeffner F, Abdulrahman B, Hickman-Davis JM, Janssen PM, Amer A, Bedwell DM, Sorscher EJ, Davis IC. 2013. Heterozygosity for the F508del mutation in the cystic fibrosis transmembrane conductance regulator anion channel attenuates influenza severity. J Infect Dis 208:780–789. doi:10.1093/infdis/jit251 23749967 PMC3733511

[37] Loeven NA, Perault AI, Cotter PA, Hodges CA, Schwartzman JD, Hampton TH, Bliska JB. 2021. The Burkholderia cenocepacia type VI secretion system effector TecA is a virulence factor in mouse models of lung infection. mBio 12:e0209821. doi:10.1128/mBio.02098-21 34579569 PMC8546862

[38] Hoen AG, Li J, Moulton LA, O’Toole GA, Housman ML, Koestler DC, Guill MF, Moore JH, Hibberd PL, Morrison HG, Sogin ML, Karagas MR, Madan JC. 2015. Associations between gut microbial colonization in early life and respiratory outcomes in cystic fibrosis. J Pediatr 167:138–47. doi:10.1016/j.jpeds.2015.02.049 25818499 PMC4674690

[39] Madan JC, Koestler DC, Stanton BA, Davidson L, Moulton LA, Housman ML, Moore JH, Guill MF, Morrison HG, Sogin ML, Hampton TH, Karagas MR, Palumbo PE, Foster JA, Hibberd PL, O’Toole GA. 2012. Serial analysis of the gut and respiratory microbiome in cystic fibrosis in infancy: interaction between intestinal and respiratory tracts and impact of nutritional exposures. mBio 3. doi:10.1128/mBio.00251-12 PMC3428694 22911969

[40] Sanville J, O’Toole GA, Madan J, Coker M. 2024. Premodulator microbiome alterations associated with postmodulator growth outcomes in pediatric cystic fibrosis: can we predict outcomes? J Pediatr Gastroenterol Nutr 79:695–704. doi:10.1002/jpn3.1235039118488

[41] Sun X, Yi Y, Yan Z, Rosen BH, Liang B, Winter MC, Evans TIA, Rotti PG, Yang Y, Gray JS, Park SY, Zhou W, Zhang Y, Moll SR, Woody L, Tran DM, Jiang L, Vonk AM, Beekman JM, Negulescu P, Van Goor F, Fiorino DF, Gibson-Corley KN, Engelhardt JF. 2019. In utero and postnatal VX-770 administration rescues multiorgan disease in a ferret model of cystic fibrosis. Sci Transl Med 11. doi:10.1126/scitranslmed.aau7531 PMC6489481 30918114

[42] Liu J, Berg AP, Wang Y, Jantarajit W, Sutcliffe KJ, Stevens EB, Cao L, Pregel MJ, Sheppard DN. 2022. A small molecule CFTR potentiator restores ATP-dependent channel gating to the cystic fibrosis mutant G551D-CFTR. Br J Pharmacol 179:1319–1337. doi:10.1111/bph.15709 34644413 PMC9304199

[43] Huttenhower lab (September 10, 2025) Kneaddata. 2025. https://github.com/biobakery/kneaddata.

[44] Blanco-Míguez A, Beghini F, Cumbo F, McIver LJ, Thompson KN, Zolfo M, Manghi P, Dubois L, Huang KD, Thomas AM, et al.. 2023. Extending and improving metagenomic taxonomic profiling with uncharacterized species using MetaPhlAn 4. Nat Biotechnol 41:1633–1644. doi:10.1038/s41587-023-01688-w 36823356 PMC10635831

[45] Beghini F, McIver LJ, Blanco-Míguez A, Dubois L, Asnicar F, Maharjan S, Mailyan A, Manghi P, Scholz M, Thomas AM, Valles-Colomer M, Weingart G, Zhang Y, Zolfo M, Huttenhower C, Franzosa EA, Segata N. n.d. Integrating taxonomic, functional, and strain-level profiling of diverse microbial communities with bioBakery 3. eLife 10. doi:10.7554/eLife.65088 PMC8096432 33944776

[46] Mallick H, Rahnavard A, McIver LJ, Ma S, Zhang Y, Nguyen LH, Tickle TL, Weingart G, Ren B, Schwager EH, Chatterjee S, Thompson KN, Wilkinson JE, Subramanian A, Lu Y, Waldron L, Paulson JN, Franzosa EA, Bravo HC, Huttenhower C. 2021. Multivariable association discovery in population-scale meta-omics studies. PLoS Comput Biol 17:e1009442. doi:10.1371/journal.pcbi.1009442 34784344 PMC8714082

